# *Boswellia* *serrata* Extract as an Antibiofilm Agent against *Candida* spp.

**DOI:** 10.3390/microorganisms10010171

**Published:** 2022-01-13

**Authors:** Petr Jaroš, Maria Vrublevskaya, Kristýna Lokočová, Jana Michailidu, Irena Kolouchová, Kateřina Demnerová

**Affiliations:** 1 Department of Biochemistry and Microbiology, University of Chemistry and Technology, 166 28 Prague, Czech Republic; petr.jaros@vscht.cz (P.J.); katerina.demnerova@vscht.cz (K.D.); 2 Department of Biotechnology, University of Chemistry and Technology, 166 28 Prague, Czech Republic; maria.vrublevskaya@vscht.cz (M.V.); jana.michailidu@vscht.cz (J.M.); irena.kolouchova@vscht.cz (I.K.)

**Keywords:** *Candida albicans*, *Candida krusei*, *Candida parapsilosis*, boswellic acid, fluconazole, biofilm

## Abstract

The use of antibiotics or antifungals to control infections caused by pathogenic microorganisms is currently insufficiently effective because of their emerging resistance. Thanks to the ability of microorganisms to form a biofilm and thus increase their resistance to administered drugs even more, modern medicine faces the task of finding novel substances to combat infections caused by them. In this regard, the effects of essential oils or plant extracts are often studied. Among the relatively neglected plants is *Boswellia serrata*, which has a high content of biologically active boswellic acids. In this study, we focused on one of the most common nosocomial infections, which are caused by *Candida* species. The most common representative is *C. albicans*, although the number of infections caused by non-albicans species has recently been increasing. We focused on the antifungal activity of *Boswellia* *serrata* extract Bioswellix against planktonic and adhering cells of *Candida albicans*, *Candida parapsilosis* and *Candida krusei.* The antifungal activity against adhering cells was further explored by determining the metabolic activity of cells (MTT) and determining the total amount of biofilm using crystal violet. Boswellic acid-containing plant extract was shown to suppress the growth of a suspension population of all tested *Candida* species. *Boswellia serrata* extract Bioswellix was most effective in inhibiting *C. albicans* biofilm formation.

## 1. Introduction

Many diseases caused by pathogenic and opportunistic pathogens are very difficult to treat with conventionally used drugs, antibiotics or antifungals, mainly due to the ability of microorganisms to form a biofilm and thus gain greater resistance to drugs [1]. Medical disciplines are not the only area where the occurrence of biofilms poses significant problems and complications [2]. Biofilms can also be found in various industries—they are involved in the corrosion of metals, reduce the efficiency of industrial heat exchangers [3] and are also a problem in the food industry [4]. We can often encounter the use of a variety of chemicals and detergents to remove the biofilm, such as hydroxides [5] or using new natural biologically active substances such as alkaloids, coumarins, saponins, tannins, quinones [6], phenols [7] and others. Natural agents also include boswellic acids, which are present in the resins of *Boswellia serata* and belong to a large group of biologically active pentacyclic triterpenes [6]. The major substance in this plant is acetyl-11-keto-ß-boswellic acid. Boswellic acids have significant activity against a number of inflammatory diseases, such as cancer, arthritis, chronic colitis, ulcerative colitis, Crohn’s disease and bronchial asthma [8,9].

New pharmacological studies have shown that in addition to anti-inflammatory effects, these compounds regulate the immune system, inhibit leukotriene synthesis, have antioxidant properties and protect the liver [10,11]. They further lower blood sugar [12] and triglycerides [13] and have a positive effect on memory [10]. They are thus used to prevent [14] and treat Alzheimer’s disease [15]. In addition to the above-mentioned health-promoting properties, boswellic acids are also monitored for significant antimicrobial activity [16]. Studies have shown the antimicrobial activity of *B. serrata* against G+ bacteria [17] and antifungal activity against *C. albicans* [18,19,20,21]. Boswellic acids have been shown in in vitro studies and animal models to act as non-competitive inhibitors of 5-lipoxygenase, inhibiting DNA, RNA and protein synthesis [22], and they significantly reduce the degradation of glycosaminoglycan [23].

Yeast species of the genus *Candida* are considered to be the most common human pathogens under clinical conditions (e.g., periodontal tissues, formation of biofilm on any artificial materials in human niches, such as catheters, dentures, etc.). Although in most cases candidiasis is caused by the yeast *C. albicans*, other pathogenic species involved in this infection, such as *C. parapsilosis*, *C. glabrata* and *C. tropicalis*, have become increasingly common in recent decades [24]. *C. parapsilosis* is generally considered to be one of the least virulent yeasts, although it is currently a common species [25]. Azoles with antifungal activity, especially fluconazole, are still a major means of therapy against *Candida* infections. Their activity consists in the inhibition of lanosterol-14-alpha-demethylase, an enzyme involved in the basic step of ergosterol biosynthesis [26]. *C. glabrata* and *C. krusei* show high levels of resistance to fluconazole and other antifungals [27]. However, clinically important antifungal agents such as fluconazole or amphotericin B, flucytosine, echinocandins [28], itraconazole [2] or ketoconazole have not been found to be as effective against *C. albicans* biofilm than against suspended cells, and the concentrations required to reduce cell metabolic activity in biofilm are 5 to 8 times higher than the corresponding minimum inhibitory concentrations (MICs) [29].

Strains of *C. albicans* resistant to azole antibiotics inhibiting ergosterol biosynthesis occur under clinical conditions due to long-term use of these antibiotics. The mechanisms of resistance of *C. albicans* biofilms to fluconazole, but also to the newly used voriconazole, depend on the ongoing phase of biofilm formation. While efflux pumps are dominant in the early stages of formation, changes in sterol composition are typical for the middle and maturation phases of the biofilm [30,31]. *C. albicans* regulates the morphological conversion of the cells to a filamentous form, which is an important virulence factor in this yeast and biofilm formation via the quorum sensing system [32]. Our study addressed the antimicrobial effect of *Boswellia serrata* extract Bioswellix and its influence on the formation and stability of the biofilm of *Candida albicans* ATCC 2091, *Candida krusei* CCM 8271 and *Candida parapsilosis* CCM 8260.

## 2. Materials and Methods

### 2.1. Antimicrobials

Bioswellix (Interpharma Slovakia), an extract from *Boswellia serrata* resin, containing 35% of the most effective pentacyclic triterpenes acetyl-β-boswellic acid (ABA) and 3-acetyl-11-keto-β-boswellic acid (AKBA), was used to test the antimicrobial activity of boswellic acids. Solutions containing boswellic acid were prepared by diluting them with dimethyl sulfoxide (DMSO), 40% ethanol or a given growth medium and were used in final concentrations ranging from 1 to 500 mg L^−1^. Fluconazole was dissolved in appropriate culture media and used at final concentrations ranging from 1 to 100 mg L^−1^.

### 2.2. Microorganisms

*C. albicans* ATCC 2091, *C. krusei* CCM 8271 and *C. parapsilosis* CCM 8260 originated from the Czech Collection of Microorganisms of Masaryk University in Brno. All strains were stored as cryopreserves in 50% glycerol at −70 °C.

### 2.3. Cultivation of Microorganisms

*C. albicans*, *C. parapsilosis* and *C. krusei* were cultured in YPD medium (20 g L^−1^ D-glucose anhydrous, Penta, Czech Republic; 20 g L^−1^ peptone, Difco Laboratories, Detroit, MI, USA; and 10 g L^−1^ yeast extract Carl Roth, Karlsruhe, Germany). Cultivation was performed for 48–72 h on an orbital shaker at 30 °C and 100 min^−1^.

### 2.4. Assay of Minimum Inhibitory Concentrations (MIC) of Antimicrobials for Suspension Yeast Populations Using a Bioscreen C Microculture Device

The determination of MIC_50_ (the lowest concentration at which 50% inhibition of suspension growth occurs after 24 h) is considered an important value expressing the susceptibility of planktonic cells to antimicrobial agents [33]. After culturing the microorganisms (see above), the inoculum was centrifuged (10 min, 10 °C, 9000× *g*) and 30 μL of inoculum (OD_600 nm_ = 0.100), and solutions of biologically active substances, were pipetted into each well of a microtiter plate (Labsystems, Helsinki, Finland). The maximum concentration of dimethyl sulfoxide or ethanol in the well did not exceed 1% and 2.5%, respectively (solvents at this concentration did not affect the culture). The wells were then filled with the appropriate complex medium to the required volume of 320 μL. Cultivation was performed in a Bioscreen C for 24 h at 30 °C.

### 2.5. Biofilm Formation

The inoculum was centrifuged (10 min, 10 °C, 9000× *g*), and its optical density was adjusted (OD_600 nm_ = 0.800). Then, 210 μL of the inoculum was transferred to a 96-well microtiter plate (TPP AG, Trasadingen, Switzerland). The cultivation of *Candida* yeasts was performed for 24 h at a temperature of 30 °C and 150 min^−1^. After that, a solution of Bioswellix or fluconazole was pipetted (the total well volume was 280 μL) to monitor the effect of biologically active substances on the initial adhesion of cells. In the case of monitoring the effect of the substances on biofilm eradication, the substances were added only after 24 h (or 48 h) of biofilm cultivation without any antimicrobials added. After washing the wells three times with 200 μL of physiological saline (0.9% NaCl (aq); Penta, Prague, Czech Republic), the culture continued in the presence of antimicrobials for another 24 h.

All experiments were performed in 8 parallels. At the end of the culture, the contents of the wells were aspirated, and the wells were washed three times with physiological saline, and then images were taken using a Cellavista apparatus (SynenTec, Elmshorn, Germany).

### 2.6. Quantification of Biofilm by Crystal Violet (CV) Staining and MTT

The total biomass content, including extracellular structures of cells adhering to the surface of the entire microtiter plate, was determined by the crystal violet staining method. Crystal violet binds to negatively charged extracellular molecules, cell surface molecules, DNA and polysaccharides as well as proteins in the extracellular matrix [34]. The procedure was taken over and modified according to Sabaeifard et al. [35]. An amount of 200 μL of a 0.1% filtered crystal violet solution (Carl Roth, Germany) was pipetted into each well, and the cells were stained for 20 min. After washing the wells twice with saline, the wells were filled with 200 μL of 96% ethanol (Penta, Czech Republic). After 10 min, 100 μL volume from each well was transferred to a microtiter plate (Gamma Group, Ostrava, Czech Republic), and the color intensity was measured at 580 nm.

As a complement to total biofilm biomass determination, the metabolic activity of the cells in biofilm was assessed using MTT. The methodology available in the current literature by Riss et al. [36] was used to determine the metabolic activity of the biofilm. At the end of the biofilm culture, the wells were washed three times with 200 μL of saline. After that, 60 μL of 57.4 mg mL^−1^ glucose solution (anhydrous glucose; Penta, Czech Republic) was pipetted into each well, plus 50 μL of a 1 g L^−1^ solution of MTT (3-(4,5-dimethyl-thiazol-2-yl)-2,5-diphenyltetrazolium bromide; Thermo Fisher Scientific, USA). Reaction was allowed to proceed for 1 h at 30 °C on an orbital shaker (150 min^−1^). Then, 100 μL of elution solution (4 parts of dimethylformamide with 6 parts of 2% (*v*/*v*) acetic acid in phosphate buffer saline) was pipetted into each well, and dodecyl sodium sulfate (160 g/L) was dissolved in this solution. After vigorous mixing (230 min^−1^; 30 min), 100 μL of the solution was taken into a 96-well plate (Gamma Group, Czech Republic) and measured spectrophotometrically at a wavelength of 570 nm.

### 2.7. Determining the Minimum Concentration of Biofilm Inhibitors (MBIC)

The minimum biofilm inhibitory concentration (MBIC_50_) was determined (procedure modified from Paldrychova et al. [37]) as the lowest concentration that causes at least 50% inhibition of the viability of formed biofilm (metabolic activity after a 24 h cultivation) in the presence of a biologically active agent. The metabolic activity was determined by the MTT (2,3-bis (2-methoxy-4-nitro5-sulfophenyl)-5-[(phenylamino) carbonyl]-2H-tetrazolium hydroxide) assay. Aliquots of 200 μL (total volume) of yeast suspension (A_600nm_ = 0.6; corresponding to 10^7^ cells per ml), obtained from the inoculum cultivation described above, were cultivated in YPD medium in the presence of the biologically active agent in a polystyrene 96-well microtiter plate (TPP AG, Trasadingen Switzerland) for 24 h at 30 °C in an orbital shaker (150 min^−1^). Concentration ranges were 0–100 mg L^−1^ for fluconazole and 0–500 mg L^−1^ for Bioswellix.

Controls without agent were also included. Each experiment was performed in eight replicates.

### 2.8. Statistical Analysis

Dixon’s Q test was performed to detect outliers in datasets obtained by the crystal violet staining and the MTT assay. The significance of the difference between control and antimicrobial-treated samples was determined by one-way analysis of variance (ANOVA) and Tukey’s HSD test.

## 3. Results and Discussion

Opportunistically pathogenic and pathogenic microorganisms have long posed a major threat to humanity because they cause a variety of diseases. Although many synthetic anti-inflammatory drugs such as steroids, non-steroidal anti-inflammatory drugs (NSAIDs) and immunosuppressants are well established for use in various types of disease, their long-term use is limited by related side effects or the development of microbial resistance to these drugs [12,38,39]. The need for safe, readily available and effective treatment of various types of diseases leads to the study of herbal medicines. Many medicinal plants have been successfully used in the treatment of inflammation since ancient times and have been used in modern medicine in pharmaceutical form [40,41]. Conventionally, antifungals such as fluconazole are used to control diseases caused by the genus *Candida*. Fluconazole is a fungistatic agent whose mechanism of action is inhibition of the activity of fungal cytochrome P450-dependent C-14α-demethylase that results in the inhibition of biosynthesis of ergosterol, which is part of the fungal cell membranes and the most important structural membrane sterol. Disruption of ergosterol synthesis damages the cell membrane of the pathogen by inhibiting its growth and replication [42]. In the human body, fluconazole is metabolized more slowly due to its chemical structure, has a lower effect on the synthesis of human sterols and is therefore suitable for systemic use. Currently, there are two main products on the Czech market containing fluconazole as an active substance—Diflucan and Mycomax [43].

Biologically active substances isolated from natural sources do not promote the development of microbial resistance and are relatively effective even at low concentrations. Medicinal plants and their chemical components are used to treat a number of inflammatory diseases. Many chemical constituents of plant origin such as alkaloids, tannins, flavonoids, terpenoids, glycosides, carotenoids and saponins are said to have anti-inflammatory properties [44,45]. Compared with synthetically prepared molecules, these substances are also effective in eradicating extracellular polymeric structures [46]. Boswellic acids, alone or in combination with antibiotics, can be an example of the search for a positive effect of natural substances [47]. Boswellic acids and their derivatives have anti-inflammatory, anti-cancer, immunomodulatory, anti-asthmatic, antibacterial and anti-rheumatic properties [48] and function as anti-inflammatory agents [49].

While there are studies, both in vivo and in vitro, on the effect of boswellic acids on tissue cells [50,51,52] or on their anti-inflammatory [53,54] or anticancer effect [55], studies on their effect on microbial strains, especially eukaryotes, are lacking.

We compared the antifungal activity of *Boswellia serrata* extract Bioswellix with fluconazole. The antimicrobial effect of boswellic acids-containing solution was investigated on three species of *Candida*, both in suspension culture and against biofilm growth. The effect of the solvent used on the antimicrobial activity of *Boswellia serrata* extract Bioswellix was also investigated (Table 1). The ineffectiveness of the substances dissolved in the medium on suspension cells was probably due to the limited solubility of the active antimicrobials in the aqueous solution [56]. A solution of *Boswellia serrata* extract Bioswellix in DMSO proved to be the most effective, and therefore further experiments were performed with this solvent.

The sensitivity of *Candida* cells to the effects of antimicrobials depends not only on the concentration of these agents but also on other factors, such as the type of medium, incubation time and temperature [57]. There are basically two media type approaches used: a richer medium such as YPD medium [58] or Sabouraud medium [59] to create favorable conditions for the growth of yeast or low glucose and nutrient media such as RPMI medium [60].

In our study, we used the same medium (YPD) for both types of culture (suspension cells and biofilm) to allow favorable growth of the microorganisms and to allow comparison of the MIC and MBIC values. As the study was aimed at initial evaluation of the potential of boswellic acid as an anticandidal agent, we used model strains and model favorable conditions to ascertain their potential, with intended follow-up studies aimed at the clinical environment (with clinical *Candida* isolates and appropriate media) and other industrial applications, such as antifouling in the food industry, which would pose a nutrient-rich environment.

Bioswellix (MIC_50_ = 20 mg L^−1^) showed the highest inhibitory effect on the suspension population of *C. albicans* ATCC 2091 (Table 1). The highest MIC_50_ value was reached for *C. krusei* CCM 8271. The MIC values of *Boswellia serrata* extract Bioswellix found in our study for various members of the genus *Candida* were in accordance with the literature since the MICs of natural substances range in the tens [61,62,63] or hundreds of mg L^−1^ [64,65,66,67,68]. Weckesser et al. [69] dealt with the effect of boswellic acids on yeasts of the genus *Candida*, but the MIC was not determined in the tested concentration range of 0.2–100 mg L^−1^. Several studies investigated the effect of boswellic acids on bacterial biofilm, such as *E. faecalis* and *S. epidermidis* [21], and the acids were also tested for their inhibitory effect on periodontal biofilm formation [70] or for suppression of skin and nail infections [71].

For the antifungal fluconazole, we found a MIC_50_ value in the range of 20–50 mg L^−1^ depending on the tested species. The efficacy of the azole-based antifungal agents used varies greatly depending on the strain tested and its specific sensitivity, and it ranges from mg L^−1^ to tens of mg L^−1^ [72,73].

The minimum inhibitory concentration of biofilm (MBIC_50_) was determined by MTT assay (Table 2), as the metabolic activity of cells of selected *Candida* strains was monitored at the same time. We did not observe any significant effect of fluconazole on the biofilm of the studied yeasts. At antifungal concentration of 100 mg L^−1^, the metabolic activity of the cells decreased only slightly (with the exception of *Candida parapsilosis* strain CCM 8260, in which the metabolic activity decreased by 50%). Maiolo et al. [72] have shown that concentrations higher than 1000 mg L^−1^ should be used to inhibit biofilm formation (MBIC).

Research on the active substances of the Boswellia plant deals with the antibiofilm effects of isolated essential oils on *Candida* yeasts rather than with boswellic acids or resin extracts of this plant. Schillaci et al. [20] confirmed the antibiofilm activity of essential oil derived from *Boswellia papyrifera* and *Boswellia rivae* on *Staphylococcus aureus*, *Staphylococcus epidermidis* and *C. albicans*. Other studies that have addressed the antimicrobial effects of essential oils on yeast of the genus *Candida* also describe their good activity against biofilms [74]. The main components of essential oils derived from the genus *Boswellia* are monoterpene substances such as α thujone, myrcene and limonene, not boswellic acids.

**Figure 1:**
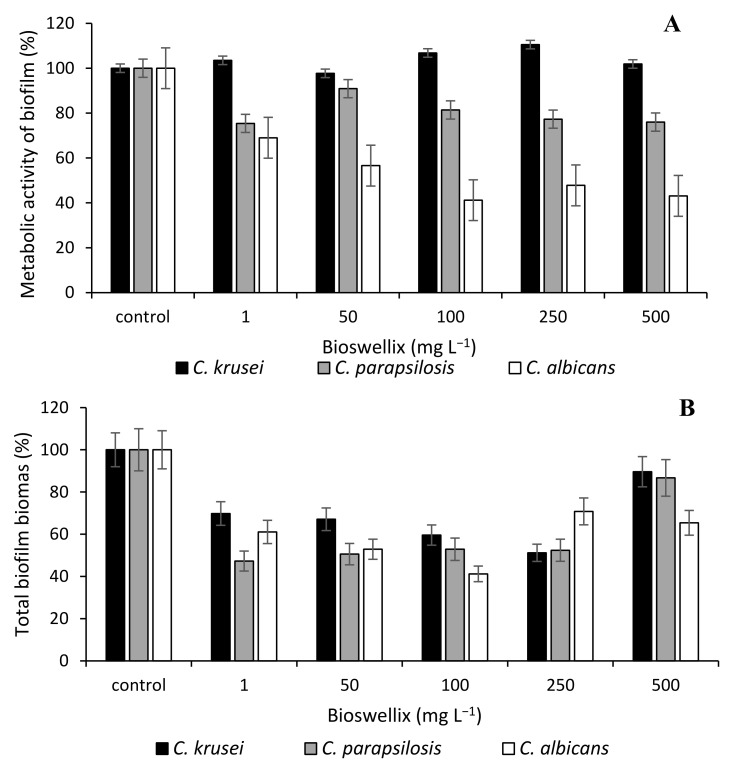
Effect of *Boswellia serrata* extract Bioswellix on *C. albicans* ATCC 2091, *C. parapsilosis* CCM 8260 and *C. krusei* CCM 8271. Biofilm metabolic activity (**A**); total biofilm biomass (**B**); control 100% (no agent). Error bars represent standard deviation.

**Figure 2:**
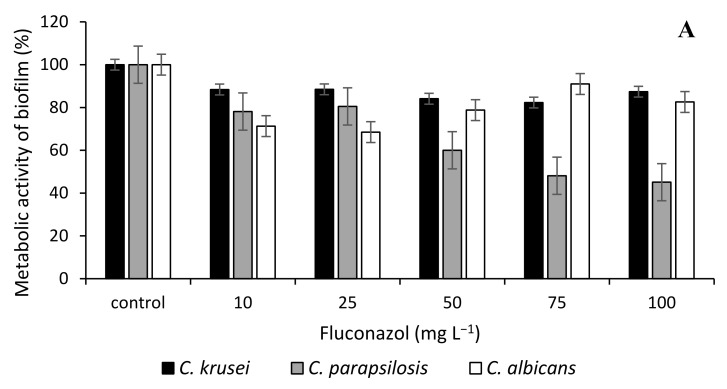

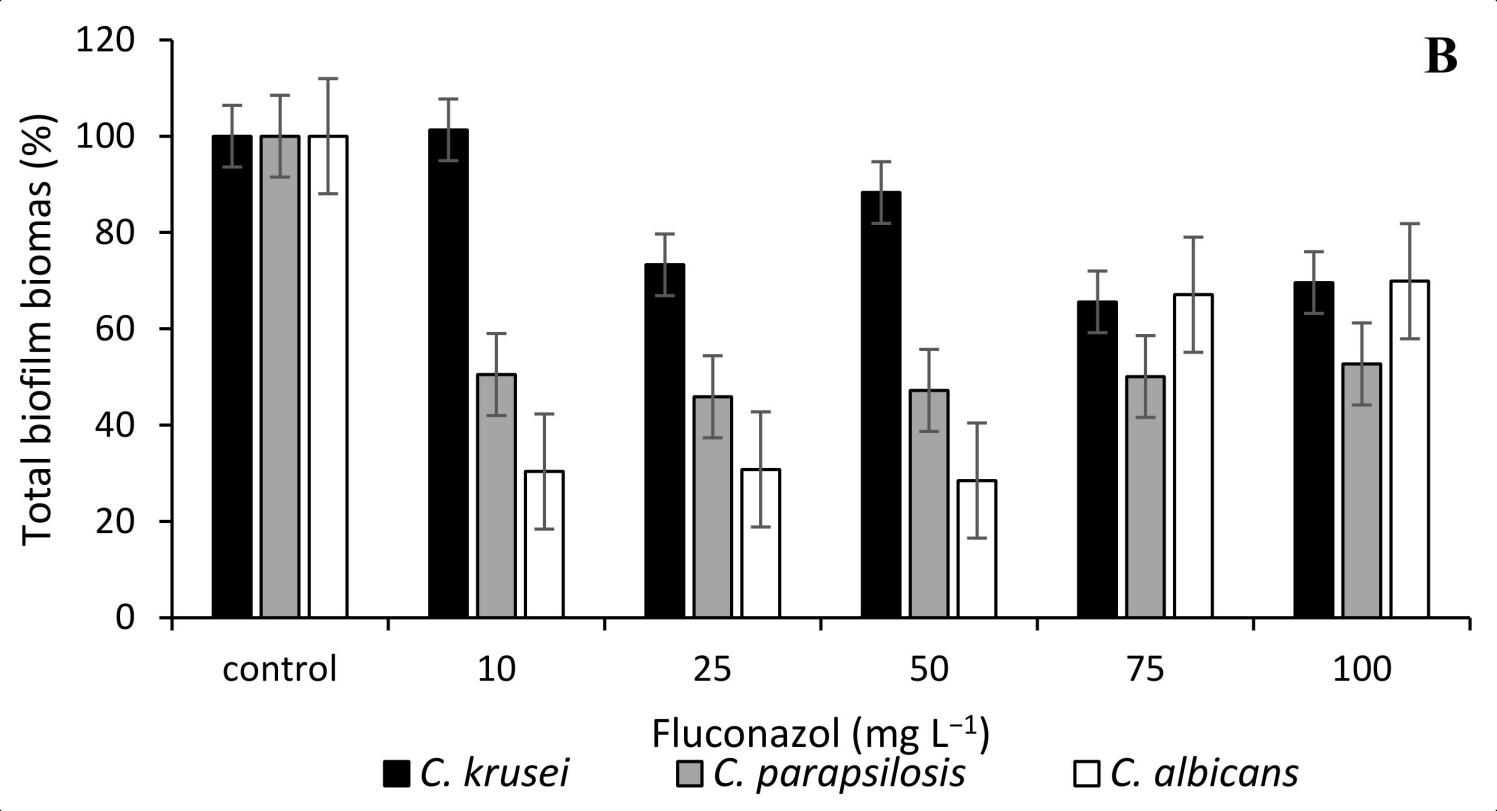
Effect of fluconazole on *C. albicans* ATCC 2091, *C. parapsilosis* CCM 8260 and *C. krusei* CCM 8271. Biofilm metabolic activity of (**A**); total biofilm biomass (**B**); control 100% (no agent). Error bars represent standard deviation.

Figure 1 and Figure 2 demonstrate the antibiofilm activity of Bioswellix and the antifungal fluconazole in terms of metabolic activity and total biomass content of *Candida*. The metabolic activity of the *Candida krusei* strain was not affected even by the concentration of 500 mg L^−1^ Bioswellix (Figure 1). Bioswellix had the greatest antibiofilm effect on the yeast *C. albicans*, in which the metabolic activity of the biofilm decreased by 30% to 60% depending on the concentration used (1–500 mg L^−1^). The decrease in the content of total biomass at all monitored concentrations also corresponded to this. The antifungal fluconazole had a negligible effect on the metabolic activity of the *C. krusei* biofilm. In general, the effectiveness of inhibiting biofilm formation was weaker for fluconazole than for boswellic acids contained in the *Boswellia serrata* extract Bioswellix (Figure 2). According to Butassi et al. [75], the increase in biofilm resistance to antifungals is associated with a significant decrease in total ergosterol content.

**Figure 3:**
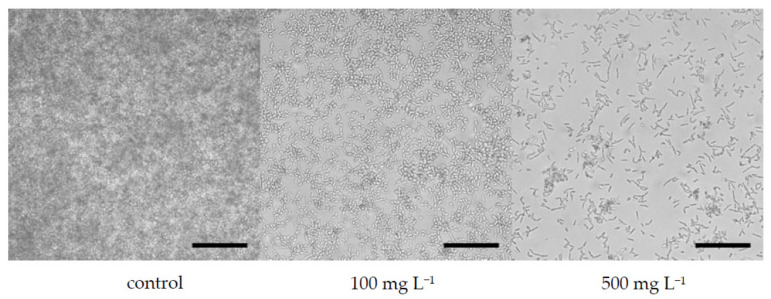
Effect of Bioswellix on *C. albicans* ATCC 2091 biofilm formation (visualized by a Cellavista device), scale bar 100 μm.

**Figure 4:**
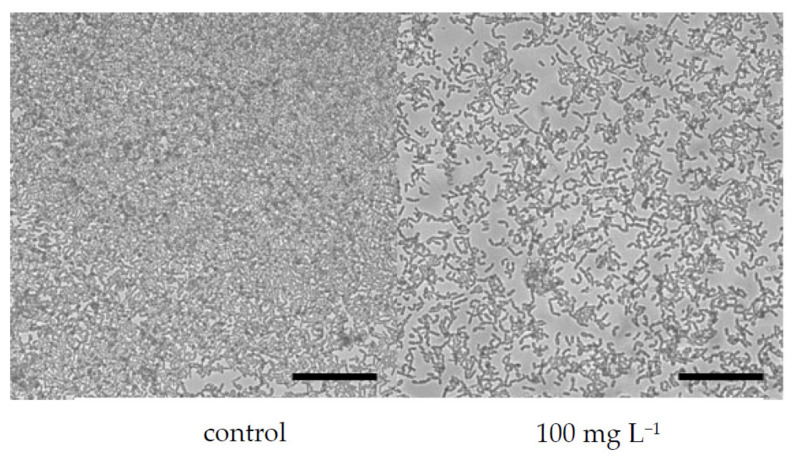
Effect of fluconazole on *C. albicans* ATCC 2091 biofilm formation (visualized by a Cellavista device), scale bar 100 μm.

The filamentous form of *C. albicans* is considered to be one of the major virulence factors that is responsible for the easier adhesion of cells to surfaces [76]. Figure 3 and Figure 4 demonstrate the morphology of *C. albicans* cells in the presence of boswellic acids contained in Bioswellix and in the presence of fluconazole (24 h old biofilm). Unfortunately, it is not possible to state that boswellic acids inhibit the virulence of *C. albicans.* Future studies should include different incubation times and a more efficacious microscopy analysis—for example, a scanning electron microscopy to conclude that *C. albicans* exposed to Bioswellix remains oval, does not change and does not undergo transition to hyphal form.

As can be seen from our results and from the research by Jenks et al. and Katragkou et al. [77,78], it is not problematic to eliminate planktonic cells (MIC) but it is problematic to eradicate cells in the form of a highly structured biofilm. Highly dangerous infections need not be caused only by *C. albicans*, which is said to be the fourth most common cause of nosocomial infections [79]; it is increasingly important to focus research on non-albicans *Candida* spp. such as *C. parapsilosis*, *C. tropicalis*, *C. krusei* and *C. auris*, which are increasingly common causes of nosocomial diseases [80,81]. It is clear from the reviews published in 2021 that the effective concentrations of various plant extracts or essential oils for inhibiting biofilm formation are in multiples of the MIC for suspension cells [57,75,82,83]. Nor do multiple MIC concentrations inhibit biofilm formation by 100%. The mechanisms of action of natural substances on *Candida* cells are often not exactly known but are associated with anti-virulence activities, anti-biofilm properties, cell wall inhibition or cell membrane synthesis and induction of cell apoptosis [68]. Due to the effects of natural substances on the *Candida* genus, it is suitable to test them together with antifungal drugs. According to current studies, these combinations have a synergistic effect, with effects on efflux pumps and on ergosterol synthesis being their most common mechanisms of action [68].

## 4. Conclusions

Antibiofilm activity of boswellic acids currently attracts considerable attention, and our study expands the issue with new knowledge. Given the results of this work, it seems that boswellic acids could act as antifungal drugs against suspension populations of *Candida* yeasts, including *Candida albicans*. Potentially, their effect in combination with antifungal drugs should be studied in search of synergy, due to the boswellic acids increasing the release of cells from biofilm.

## Figures and Tables

**Table 1:** Minimum inhibitory concentrations (MIC_50_) of Bioswellix and fluconazole for *C. albicans* ATCC 2091, *C. parapsilosis* CCM 8260 and *C. krusei* CCM 8271.

MIC_50_ (mg L^−1^)	Microorganism		
	*C. parapsilosis* CCM 8260	*C. krusei* CCM 8271	*C. albicans* ATCC 2091
Bioswellix (EtOH)	200	280	250
Bioswellix (DMSO)	80	120	20
Bioswellix (medium)	500 ^†^	500 ^†^	500 ^†^
Fluconazole	20	35	50

^†^ has not been determined up to the above concentration.

**Table 2:** Minimum biofilm inhibitor concentrations (MBIC_50_) of Bioswellix and fluconazole for *C. albicans* ATCC 2091, *C. parapsilosis* CCM 8260 and *C. krusei* CCM 8271.

MBIC_50_ (mg L^−1^)	Microorganism		
	*C. parapsilosis* CCM 8260	*C. krusei* CCM 8271	*C. albicans* ATCC 2091
Bioswellix (DMSO)	500 ^†^	500 ^†^	100
Fluconazole	75	100 ^†^	100 ^†^

^†^ has not been determined up to the above concentration.

## Data Availability

All relevant data are contained within this article.
